# Editorial: Big Data Management in Industry 4.0

**DOI:** 10.3389/fdata.2021.788491

**Published:** 2021-11-25

**Authors:** Donatella Firmani, Francesco Leotta, Federica Mandreoli, Massimo Mecella

**Affiliations:** ^1^ Department of Statistical Sciences, Sapienza Università di Roma, Rome, Italy; ^2^ Department of Computer, Control and Management Engineering, Sapienza Università di Roma, Rome, Italy; ^3^ Department of Physics, Informatics and Mathematics, Università degli Studi di Modena e Reggio Emilia, Modena, Italy

**Keywords:** big data management, smart manufactoring, industry 4.0, digital twin (DT), predictive maintainance

Industry 4.0 represents the digital evolution of manufacturing, where emerging technologies such as Industrial Internet of Things (IIoT) Sisinni et al. (2018), data analytics, artificial intelligence, cloud computing and cyber physical systems Lee et al. (2018) enable operation of industries in a flexible, efficient, and green way.

As such, it is of the utmost importance to be able to manage and analyse the huge amount of data originating from the deployed sensors and services, which can be classified as Big Data from the point of view of volume, velocity, variety, veracity, and value (5 V s).

Digital factory is a fundamental notion within the Industry 4.0 domain: it comprises a multi-layered integration of the factory activities, enabling the product lifecycle stakeholders to collaborate through the use of software solutions. A digital factory can expand outside the company boundaries and offers the opportunity to collaborate on business processes affecting the whole supply chain Bicocchi et al. (2019).

Digital Twin (DT) is one of the most prominent technologies for realizing digital factories and Industry 4.0 Tao et al. (2018). A DT is a digital representation of a physical asset (e.g., a machine, a partner organization, a product), which can be employed for *1*) querying its status, *2*) issuing commands, *3*) receiving streaming data, *4*) performing prediction tasks, and *5*) simulating the effect of specific usage conditions. A digital factory can be represented with the DTs involved in the manufacturing process. The result can be thought of as an heterogeneous *data space*, populated by the DTs, and a coordination entity in charge of pursuing specific tasks Catarci et al. (2019). Data coming from the DTs can be used by the coordination entity for a wide variety of tasks, including monitoring the status of the process, adapting to sudden or long term changes, improving the process according to specific Key Performance Indicators (KPI), and predicting the evolution of all the assets involved in the manufacturing process.

The digital factory data space can include structured, semi-structured and unstructured data with different schemata, vocabularies, and data access technologies. Due to such heterogeneity and to the nature of the involved data, only a small fraction of the existing data management techniques can be directly applied in a digital factory data space. As a consequence, existing approaches struggle to capture the production goal semantic and mainly focus on specific interoperability aspects. Furthermore, existing data modeling frameworks for DTs (either commercial or open-source) do not provide full support for simulation and prediction.

The research topic “Big Data Management in Industry 4.0” includes contributions in several of the aforementioned areas.

Authors in Bousdekis and Mentzas (2021) discuss the challenge of integrating traditional enterprise software solutions (e.g., Enterprise Resource Planning systems (ERPs) and Computerized Maintenance Management System (CMMS)) with Big Data analysis and Business Process Mining techniques. As a case study, authors show a predictive maintenance platform, for the steel manufacturing industry, that was developed in accordance with the proposed architectural framework.

Predictive maintenance is also the final goal of the contribution presented in Sang et al. (2021). Here the main focus is not on the architecture, but instead a deep learning based toolbox for predictive maintenance, and in particular to estimate the Remaining Useful Life (RUL) of assets. The effectiveness of the proposed solution is demonstrated using a real-world industrial case.

A typical challenge of data management in smart manufacturing is ensuring that the quality of the data is good enough to allow for reliable analysis. Authors in Ramalli et al. (2021) present an innovative approach to data quality management from the chemical engineering domain, based on an available prototype of a scientific framework.

Authors in Albers et al. (2021) discuss the challenges encountered by applying data analysis techniques in a dynamic environment such as a smart factory. In this case, processing pipelines must continuously evolve in terms of configuration parameters. In particular, automated planning is employed to find the best configuration according to the context.

The topics covered in this special issue identify data analytics and data quality management as crucial tools for manufacturing chains with *zero down time*. The authors agree that implementing such tools in the Industry 4.0 domain raises different challenges, both from the architectural and methodological perspective. The data management pipelines described in the above works include for instance Business Process Mining functionalities, Deep Learning tools for predictive maintenance, scientific data modeling and dynamic configuration options, thus enabling a wider range of smart manufacturing applications with respect to traditional Big Data frameworks.

Predictive maintenance is envisioned by the authors of this special issue as one of the main down-stream tasks of an Industry 4.0 data management pipeline. This clearly defines the current and future research directions towards the new era of industry, denominated Industry 5.0, that will be sustainable, human-centric, and resilient. Predicting the behaviour of physical assets and anticipating their failure is indeed identified as one of the core abilities of future smart manufacturing pipelines, and the key ingredient for better human-machine collaboration, higher energy saving, and the deployment of more robust reconciliation strategies.
